# Phylogenomic diversity of *Vibrio* species and other Gammaproteobacteria isolated from Pacific oysters (*Crassostrea gigas*) during a summer mortality outbreak

**DOI:** 10.1099/mgen.0.000883

**Published:** 2022-12-07

**Authors:** Paul J. Worden, Daniel R. Bogema, Melinda L. Micallef, Jeffrey Go, Ania T. Deutscher, Maurizio Labbate, Timothy J. Green, William L. King, Michael Liu, Justin R. Seymour, Cheryl Jenkins

**Affiliations:** ^1^​ NSW Department of Primary Industries, Elizabeth Macarthur Agricultural Institute, Woodbridge Rd, Menangle, NSW 2568; ^2^​ School of Life Sciences, Faculty of Science, University of Technology Sydney, Sydney, NSW, Australia; ^3^​ Centre for Shellfish Research, Vancouver Island University, Nanaimo, British Columbia,, Canada; ^4^​ Department of Plant Pathology and Environmental MIcrobiology, The Pennsylvania State University, University Park, PA 16802, USA; ^5^​ iThree Institute, University of Technology Sydney, Building 4, 745 Harris Street, Broadway, Ultimo, NSW, 2007; ^6^​ Climate Change Cluster, University of Technology Sydney, Ultimo, NSW, 2007

**Keywords:** accessory genome, average nucleotide identity, core genome, Crassostrea gigas, microbial pathogens, microbiota, Pacific oyster, pan genome, phylogenetic tree, phylogenetics, summer mortality, whole genome sequencing, Vibrio

## Abstract

The Pacific oyster (PO), *Crassostrea gigas*, is an important commercial marine species but periodically experiences large stock losses due to disease events known as summer mortality. Summer mortality has been linked to environmental perturbations and numerous viral and bacterial agents, indicating this disease is multifactorial in nature. In 2013 and 2014, several summer mortality events occurred within the Port Stephens estuary (NSW, Australia). Extensive culture and molecular-based investigations were undertaken and several potentially pathogenic *Vibrio* species were identified. To improve species identification and genomically characterise isolates obtained from this outbreak, whole-genome sequencing (WGS) and subsequent genomic analyses were performed on 48 bacterial isolates, as well as a further nine isolates from other summer mortality studies using the same batch of juveniles. Average nucleotide identity (ANI) identified most isolates to the species level and included members of the *Photobacterium *, *Pseudoalteromonas *, *Shewanella* and *Vibrio* genera, with *Vibrio* species making up more than two-thirds of all species identified. Construction of a phylogenomic tree, ANI analysis, and pan-genome analysis of the 57 isolates represents the most comprehensive culture-based phylogenomic survey of *Vibrios* during a PO summer mortality event in Australian waters and revealed large genomic diversity in many of the identified species. Our analysis revealed limited and inconsistent associations between isolate species and their geographical origins, or host health status. Together with ANI and pan-genome results, these inconsistencies suggest that to determine the role that microbes may have in Pacific oyster summer mortality events, isolate identification must be at the taxonomic level of strain. Our WGS data (specifically, the accessory genomes) differentiated bacterial strains, and coupled with associated metadata, highlight the possibility of predicting a strain’s environmental niche and level of pathogenicity.

## Data Summary

All genome assemblies mentioned here have been submitted to NCBI under BioProject accession number PRJNA795272. The authors confirm that all supporting data, code, and protocols have been provided within the article or through supplementary data files.

**Impact Statement**

Summer mortality in Pacific oysters can have a devastating effect on this industry, and although no definitive cause has been discovered, there is increasing evidence that summer mortality is a multifactorial disease which somehow includes elements of the oyster microbiome. This study compares the relatedness of isolate genomes taken from Pacific oysters suffering summer mortality (and some controls) and dates and locations around Port Stephens. We identified not only a diverse set of species belonging to the Gammaproteobacteria class (including many *Vibrios*), but for species with three or more isolates a high genetic diversity was also seemingly apparent.

## Introduction

Of all the oysters commercially grown worldwide, the Pacific oyster (PO), *Crassostrea gigas*, is the most geographically distributed and economically important oyster. It is the only oyster farmed on all inhabited continents, with a total harvest weight in 2017 of 637 742 metric tons, valued at just over $US1.24 billion [1]. Due to a combination of accidental and deliberate dispersal of POs, cultivation of this species has increased markedly since the end of World War II in terms of both tonnage and locations farmed [2, 3]. However, expanding production has been accompanied by a simultaneous worldwide increase in disease events in *C. gigas,* including periodic disease outbreaks occurring during warmer months, known as summer mortality (SM). Despite decades of research, no definitive cause of SM in POs has been determined [4, 5]. Biotic stresses such as viral and bacterial pathogens [6], as well as genetic [7] and reproductive factors [8, 9] have all been implicated, as have many abiotic stresses, especially heat stress, low salinity (high rainfall), ocean acidification (pH change), low dissolved oxygen, and pollution [10–13]. On balance, although a definitive cause remains elusive, the sum of evidence suggests that SM is caused by a multifactorial combination of biotic and abiotic stresses [4, 14–17].

Pacific oyster mortality syndrome (POMS) is another important disease of *C. gigas* that is often confused with or mislabelled as SM, both for historical reasons and because it also occurs during warmer weather. In general SM tends to affect older POs experiencing their second summer, while POMS usually affects spat and juveniles. Moreover, POMS is caused by a virus - an ostreid herpesvirus (OsHV-1 and its variants). Herpes-like viruses have been observed during PO SM events since 1991 [18, 19], and after the characterisation of an ostreid herpesvirus (OsHV-1) in 1999 [20, 21], OsHV-1 and its variants were observed in a number of Pacific oyster SM events worldwide, including Australia [22–26]. Moreover, during many of these events, high bacterial loads of one or two *Vibrio* species (including *V. splendidus *, *V. aesturianus*, *V. tubiashii *, *V. harveyi *), were detected alongside infection with OsHV-1 [6, 25, 27]. It has also been observed that the presence of certain pathogenic vibrios is required for full pathogenicity of OsHV-1 [28]. While de Lorgeril *et al*. [29] observed that PO immune suppression by the OsHV-1 virus leads to the PO being overwhelmed by opportunistic bacteria, in another study the pathogenicity of virulent *Vibrio* strains at low doses was enhanced by the presence of avirulent *Vibrios* [30].

Worldwide, there have also been many SM events where putative bacterial pathogens were observed to be the only etiological cause of disease. During the 1950s and 60s, nocardiosis - a disease caused by the bacteria *Nocardia crassostreae,* was observed during summer mortalities of POs in Japan, Canada, and the USA [31–34]. *Ricksettsia*-like bacteria have also been implicated in PO SM [35]. Yet, species within the *Vibrio* genus have most often been associated with reports of SM and POMS [27, 29]. Larval vibriosis (or bacillary necrosis) has been observed in hatcheries, often resulting in more than 90 % mortality [36]. PO mortality from vibriosis has also been observed in oyster spat [37, 38] and adults [39]. Indeed, for all *C. gigas* life stages several putative *Vibrio* pathogens have been identified, including *V. alginolyticus *, *V. tubiashii *, *V. anguillarum *, *V. parahaemolyticus* and *V. harveyi* [4, 30, 40, 41]. Investigations of bacterial community composition during SM disease events have also identified significant increases in *Vibrio* relative abundance [42–44].

Several SM events, where high temperatures appeared to play a role, occurred between January 2013 and January 2014 within the Port Stephens (PS) estuary in NSW [4]. These events were investigated in three stages (see Methods) which are, briefly: 1) diagnostic submissions of diseased POs, 2) a structured survey of selected SM-affected leases and control sites, and 3) a challenge trial. In total this 2013–2014 study was significant because no OsHV-1 or variants were detected in association with mortalities, and the bacterial isolates obtained represented a wide range of species, many of which were identified as *Vibrios* by characterization of the 16S rRNA taxonomic maker gene. A 16S-based microbiome study of the surveyed POs also revealed changes in relative abundance of certain bacterial groups including rare OTUs belonging to *V. harveyi* and another unknown *Vibrio* species [45]. To further investigate which species and strains may have been involved in this outbreak we used whole-genome sequencing to characterise 48 isolates from affected and unaffected POs and as a comparison, a further nine isolates originating from the same stock of juvenile POs.

## Methods

### Definition of SM-affected and SM-unaffected

Multiple SM events in POs occurred in Port Stephens between January 2013 and January 2014 that were investigated in three stages. Isolates obtained from SM-affected POs were acquired from the first two stages: 1) diagnostic testing of oysters from nine leases suffering mortalities, 2) a structured survey of ten affected sites and four unaffected sites around Port Stephens. Isolates from SM-unaffected hosts were obtained only from the second stage. Isolates from the third stage challenge trials as described in Go *et al*. [4] were not used in this work. All SM-affected sites suffered mortalities above the background rate, and the SM-affected POs from which isolates were derived also displayed signs of disease (gaping, high bacterial load). SM-unaffected POs from which isolates were derived were from control sites with no mortalities above the background rate and appeared healthy.

### Bacterial isolates used in this study

**Fig. 1.**
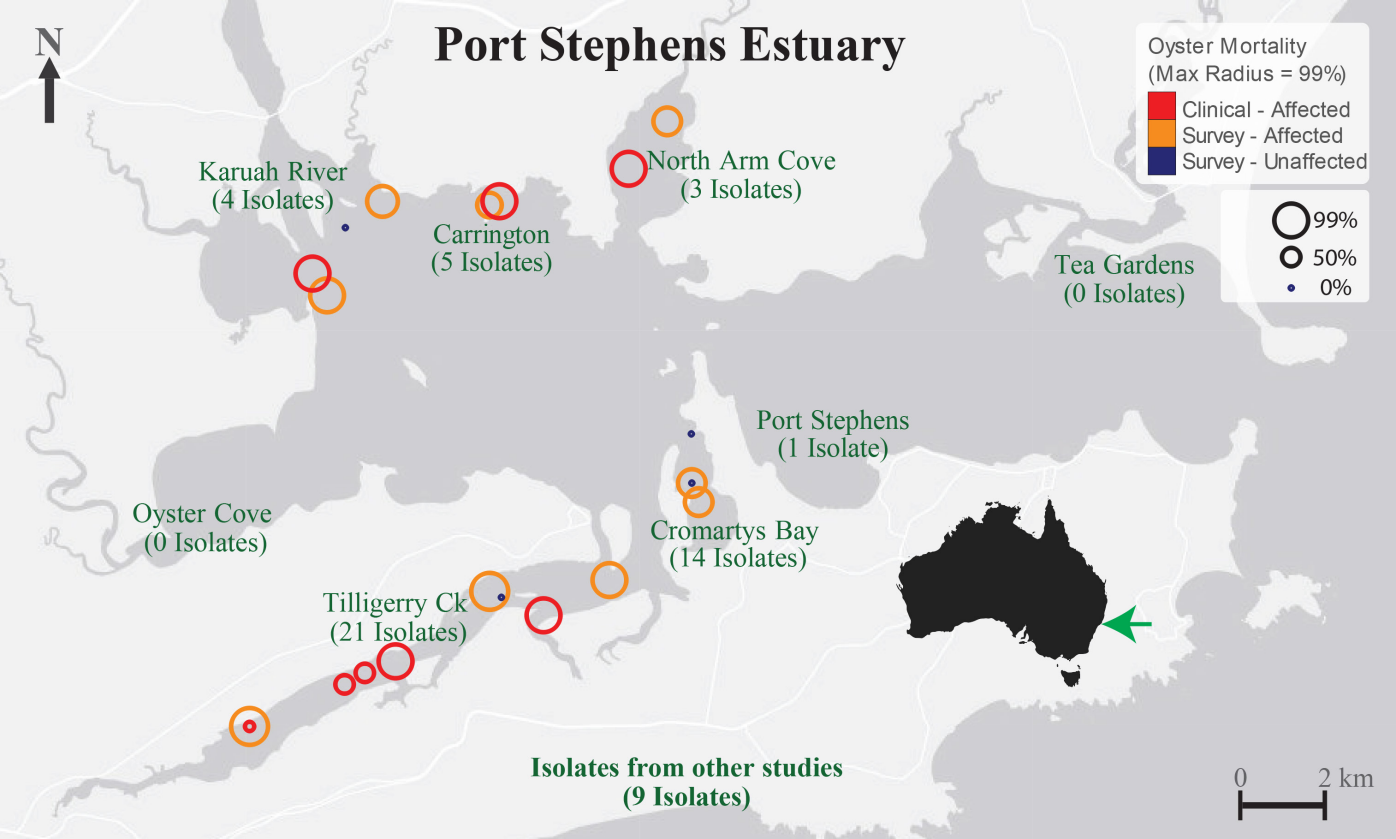
Locations of the host PO sample sites from which the bacterial isolates were derived, with POs sampled during the SM outbreak of January 2013 to January 2014. Red circles represent the sites of diagnostic submissions, while orange and blue circles represent sites of the structured survey that included both affected and unaffected POs, respectively.

The 48 isolates obtained during the structured survey in 2013–2014 were derived from PO hosts grown at six locations within the PS estuary, including several locations with multiple sites (Fig. 1). At Tilligerry creek, nine sites (including two control sites) were sampled yielding 21 isolates, while at Cromartys Bay, four locations (two controls) were sampled resulting in 13 isolates. Tilligerry Creek and Cromartys Bay are close to more urbanised parts of the PS estuary, with the Tilligerry Creek catchment also containing abandoned mines. Although the greatest number of isolates originated from Tilligerry Creek, the proportion of isolates per affected site was greatest for Cromartys Bay with a significantly higher bacterial abundance in affected oysters relative to unaffected POs [4]. A further nine isolates were also obtained from the same stock of juvenile POs. Six of the nine originated from other diagnostic cases occurring during the 2013–2014 SM event, following translocation from Port Stephens [4]. Another isolate was from a previous Port Stephens mortality event (2008), and two (*V. harveyi,* PS05 and PS09) were derived from a study in Oyster Cove, Port Stephens, investigating heat stress in POs [42].

### Nucleic acid extraction and whole-genome sequencing

DNA was extracted from isolates using the DNeasy Blood and Tissue kit (Qiagen) according to instructions from the manufacturer. The Nextera system (Illumina) involving Tagmentation of genomic DNA and PCR amplification was performed on each sample. The sequencing libraries were pooled and normalized using bead size selection (SPRI beads from Beckman Coulter). The Agilent 2100 Bioanalyzer, with the High Sensitivity DNA kit was used to quantitate the pooled library before loading. Whole-genome sequencing was performed on the Illumina HiSeq and MiSeq instruments. Paired-end 250 nt reads were generated using MiSeq V2 chemistry while paired-end 150 nt reads were generated using the HiSeq2500 system.

### Genome assembly and annotation

Genome assembly was achieved by running the Illumina raw reads through the A5-miseq pipeline (version 20160825) as described in Coil *et al*. [46]. The ‘RAST’ (https://rast.nmpdr.org/) and Prokka v1.12 pipelines were used to perform genome annotation [47, 48]. Quality of the isolate genome assemblies was examined using Quast (https://github.com/ablab/quast).

### Detection of potential plasmids in *V. harveyi* isolates

Alignments of the *V. harveyi* isolate genomes (M14-00197 and M14-00480) were performed by the progressive Mauve [49] plugin for the Geneious software v. 5.3.6 [50], using default values. An approximately 86 Kbp DNA fragment was found to be missing from M14-00480, with the corresponding additional DNA sequence in M14-00197 checked for plasmid homology using BLASTn-Megablast searches [51] with default parameters. The 86 Kbp fragment was found to be homologous to several plasmids, and later confirmed to be circular by PCR using custom primers amplifying outwards from each end of the fragment (Table S1). Another 86 Kbp plasmid was detected in PS05, using a custom blast database of the 86 Kbp putative plasmid found in *V. harveyi* M14-00197 that was constructed from the NCBI command-line application makeblastdb.

### Isolate species identification by WGS and ANI

Isolate genomes and a subset of reference and representative refseq genomes within the Gammaproteobacteria were compared against each other, using an ANI cut off level of 95 % or greater to identify which bacterial species each isolate belonged to [52, 53]. All genomes were downloaded from NCBI, and their average nucleotide identity (ANI) compared against those of the unknown isolates using the bioinformatic packages FastANI and Pyani. FastANI is a whole-genome similarity estimation utility used for high throughput parsing of ANI comparisons [54], whereas Pyani [55] is a python three module (version 0.2.10, available at https://github.com/widdowquinn/pyani) that is slower but incorporates a more comprehensive algorithm. For pyani the ANIm method of calculating percent identity was used in which input sequences were aligned with MUMmer [56]. Isolates were identified by species if they were found to have an ANI identity of 95 % or greater to previously identified species of published bacterial genomes downloaded from NCBI. Additional ANI visualisations were performed using a custom R-script based on the ‘pheatmap’ R-package (https://github.com/raivokolde/pheatmap).

### 16S identification of isolate species unidentified by WGS and ANI

Isolate species that could not be identified by ANI comparisons against reference genomes from NCBI (ANI ≤95 %), were instead similarly compared according to their 16S rRNA gene sequences. The 16S rRNA gene was identified within WGS isolate genomes using the ContEst16S web service (https://www.ezbiocloud.net/tools/contest16s), described in [45]. Then BLASTn, using the megablast search with default settings, was performed on the extracted 16S gene from each sample and the species identification accepted if the percent identity was greater or equal to 98.65 %. All 16S reference strains were type strains for each species. The 98.65 % (percent identity) species cut-off was chosen based on Kim *et al*. [57], who added to prior work, especially Stackebrandt and Ebers [58] and Meier-Kolthoff *et al*. [59].

### Construction of a phylogenomic tree

After the identification of isolates by genus or species, a phylogenomic tree was constructed by genomic alignments of core genes from each isolate using the phylosift suite (version 20141126; available at https://phylosift.wordpress.com/ [60). After phylogenomic alignments of core bacterial genes by phylosift [60] a newick formatted file was produced using FastTree-20.1.10 [61]. Final visualisation and annotation using the newick file was then implemented by the web based bioinformatic tool: Interactive tree of life (iTOL) (https://itol.embl.de/). NCBI bacterial genomes were also downloaded and used in combination with isolate genomes to generate many phylogenomic trees and confirm species identities determined by ANI. Initial confirmatory phylogenomic trees consisted of all reference and representative bacterial genomes along with the isolate genomes. These initial phylogenomic trees were then iterated on with genus and then species-specific reference genomes.

### Pan-genome examination

The pan-genome of all sequenced isolates was examined using Roary v3.13.0 [62]. Roary was executed with default options. Gene presence/absence plots were generated using roary_plots.py (https://github.com/sanger-pathogens/Roary/tree/master/contrib/roary_plots). Other pan-genome plots not presented here were generated using the web-based tool ‘Phandango’ (http://jameshadfield.github.io/phandango/#/), as described in Hadfield *et al*. [63]. Custom R-scripts were used with Roary output to examine gene differences between isolates obtained from SM affected POs versus SM unaffected POs.

### Gene ontology

The functional profile of selected accessory genomes was also examined. Namely the accessory genomes of *V. brasiliensis *, *V. harveyi* and *V. mediterranei* that represented a mix of potential pathogens and commensal isolates, as well as *V. parahaemolyticus* and *V. mediterranei* whose isolates were all from either affected or unaffected oysters respectively. Initially, gene ontology from all five pan-genomes were examined using shinyGO (http://bioinformatics.sdstate.edu/go/), or manually using gene ontology data from the Uniprot knowledge base (https://www.uniprot.org/), the GO consortium (http://geneontology.org/), and quickGO (https://www.ebi.ac.uk/QuickGO/annotations). For gene ontology analysis the R-package seqinr was used to construct a fasta file consisting of a gene subset representing the accessory genomes of *V. parahaemolyticus, V. chagasii*, *V. brasiliensis *, *V. harveyi *, and *V. mediterranei* from the initial fasta output of Roary. The fasta file of the accessory genome for each of the five species listed were then subjected to Diamond-Blastx [64] database searches using the Uniprot protein database and a list of gene identifiers obtained. Strict subsets of accessory genes were also concatenated into fasta files, with each file representing genes that were present in one isolate set of a species while completely absent in the other isolate set. For each species the two sets differed by locations of origin (* V. chagasii *), or potential pathogenicity (* V. brasiliensis *, *V. harveyi *, and *V. mediterranei *). Excluding *V. brasiliensis,* the strict gene subset for *V. chagasii, V. harveyi*, and *V. mediterranei *) resolved down to a comparison between one variable: location (* V. chagasii *) or suspected pathogenicity (* V. harveyi* and *V. mediterranei *). The two *V. harveyi* isolate sets were based on presence or absence of an 86 Kbp plasmid The gene ontology terms were obtained by gene identifiers from the blastx search being input into the Uniprot website (https://www.uniprot.org/uploadlists/). For all gene ontologies examined, generic summaries (GO-SLIMS) of gene ontology terms were obtained from quickGO or the GO-SLIMS tool at AgBase (https://agbase.arizona.edu/index.html), described in McCarthy *et al*. [65]. Gene Ontology summary of terms was performed by Revigo (http://revigo.irb.hr/), described by Supek *et al*. [66], using default settings – with the exception of the list size being set to small. CirGO (https://github.com/IrinaVKuznetsova/CirGO) was used for additional summation and visualisation of the enriched gene ontology data [67].

### Mobile genetic elements (MGEs)

*Vibrio harveyi* isolate pairs M14-00197 and M14-00480, as well as *V. harveyi* PS05 and PS09 were manually analysed and mapped for mobile genetic elements (MGEs) via several bioinformatic programmes and web services. The genomes were investigated for insertion sequence elements (ISEs) via ISEScan [68]. Prophage sequences were searched for using PhiSpy [69] as well as PHASTER [70]. Genomic islands were searched for using IslandViewer4 [71]. CRISPR sequences were detected using Geneious [50]. All MGEs mentioned above were mapped to the four *V. harveyi* genomes also through Geneious (https://www.geneious.com/). In contrast, all isolates were bioinformatically scanned for antibiotic resistance using Abricate [72] [https://github.com/tseemann/abricate] and the virulence factor database [73] using the full dataset of verified and potential virulence factors.

## Results

For the 57 isolates sequenced the median total length of genome assemblies was 5220 Kbp, ranging from a maximum of 6570 Kbp to a minimum of 3700 Kbp. The number of contigs per assembly ranged from 2742 (single extreme outlier) down to 16, with a median of 70, while the median N50 was around 196 Kbp within a range of 4640 Kbp to 2190 Kbp. The percent GC of all isolates ranged between 40–53 with a median of 45. See Table S2 of the supplementary material for the quality measures of the isolate as determined by Quast.

**Fig. 2.**
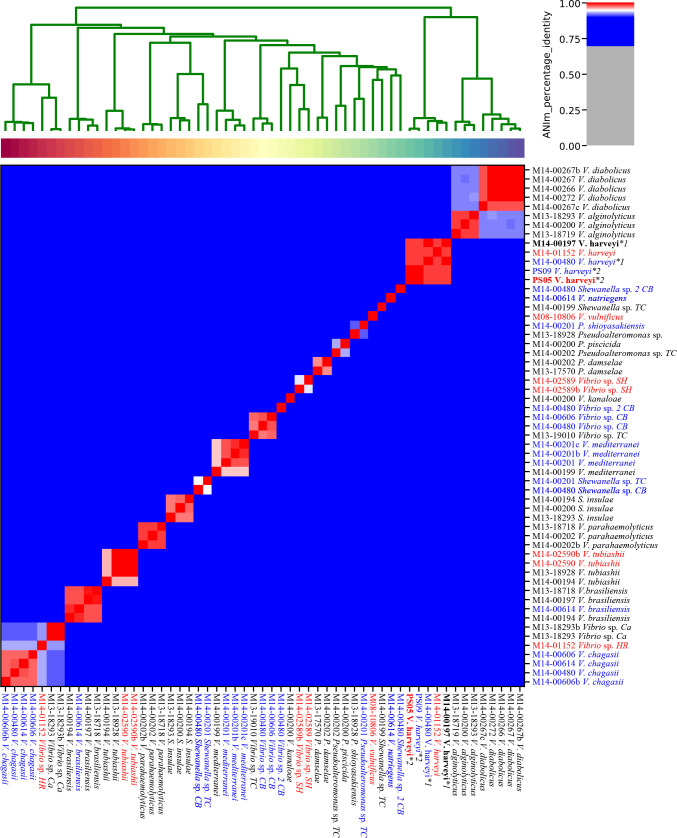
Clustering of isolates by comparing average nucleotide identity (ANI) between genomes using the MUMmer ultra-rapid aligning tool. The shades of red on the heatmap denote an ANI≥95 % (same species). The isolate names marked in red text were not part of the 2013–2014 study, while the isolates in blue text originate from SM-unaffected POs within Port Stephens with black text representing SM-affected POs.The single asterisk denotes two sets of two *V. harveyi* isolates, each set being essentially two clones differentiated almost entirely by the presence or absence of an 86 Kbp DNA fragment (suspected plasmid). Isolates PS05 and M14-00197 containing the 86 Kbp plasmid are in bold text. The numbers preceding the species or genus name represent the submission number of the oyster batch received. It should be noted that occasionally multiple isolates originated from a single oyster.

The WGS results were used to identify species, and to study gross genomic and functional differences between isolates of the same species in terms of their geographic origin, or their potential involvement in pathogenicity (i.e., possibly pathogenic isolates if obtained from SM-affected POs). Species identification of isolates was achieved by comparing the ANI of each isolate with reference bacterial genomes downloaded from NCBI, especially type strains if they were present. ANI results from all 57 isolates are summarised in Fig. 2, and identifications to type strains in Table 1. Out of a total of 15 species identified from WGS, the majority of species (11) were identified as belonging to the *Vibrio* genus, as expected from the results reported in Go *et al*. [4]. The remainder were identified as belonging to the genera *Shewanella *, *Photobacterium *, and *Pseudoalteromonas *. Isolates categorised as *Vibrio chagasii *, *V. kanaloae *, *V. mediterranei *, *V. brasiliensis *, *V. tubiashii *, *V. vulnificus *, *V. harveyi *, *V. parahaemolyticus *, *V. natriegens *, and *V. alginolyticus* all had ANI matches to their respective type strains whose genomes had been fully sequenced (see Table 1). Similarly, *Shewanella insulae *, *Pseudoalteromonas piscicida* and *P. shioyasakiensis* isolates were identified with matches to their WGS type strains. The *V. diabolicus* isolates were identified with matches to 19 reference genomes (non-type strains), as the type strain had no fully sequenced genomes. Thus, of the 57 isolates, 42 were identified to the species level with the remaining 15 only identified to the taxonomic level of genus. In total ANI comparisons of isolate genomes to those from the following taxonomic orders were made: *Oceanospirillales *, *Alteromonadales* (containing *Pseudoalteromonas* and *Shewanella* genera), *Vibrionales* (containing *Vibrio* and *Photobacterium* genera), and *Aeromonadales *.

**Table 1.** Identified species of isolates and their type-strain/s

Species	Type strain/s	ANI≥95 % Identity to fully sequenced type strain/s
* Pseudoalteromonas piscicida *	ATCC 15057, JCM 20779	Yes
* Pseudoalteromonas shioyasakiensis *	JCM 18891	Yes
* Shewanella insulae *	JBTF-M18	Yes
* Photobacterium damselae *	CIP 102761, NCTC11647, ATCC 33539	Yes
* Vibrio chagasii *	LMG 21353, CCUG 48643	Yes
* Vibrio kanaloae *	CCUG 56968	Yes
* Vibrio mediterranei *	UVEG, NBRC15635	Yes
* Vibrio brasiliensis *	LMG 20546	Yes
* Vibrio tubiashii *	ATCC 19109(2014)	Yes
* Vibrio vulnificus *	ATCC 27562(2017), NCTC13647, NBRC 15645(2017)	Yes
* Vibrio harveyi *	FDAARGOS_109, NCTC12970, NBRC 15634, ATCC 14126	Yes
* Vibrio parahaemolyticus *	FDAARGOS_115, NCTC10903, NBRC 12711, ATCC 17802(2017), CAIM 320	Yes
* Vibrio natriegens *	ATCC 14048(2016), CCUG 16371, NBRC 15636, DSM 759	Yes
* Vibrio alginolyticus *	FDAARGOS_97, ATCC 17749, NCTC12160, NBRC 15630	Yes
* Vibrio diabolicus *	HE800, 16S sequence only	No WGS Data*

*No WGS assembly currently exists in the NCBI database for the type-strain.

For the 15 isolates unidentified to species level, 16S rRNA gene comparisons were also employed as an identification tool, using 98.65 % sequence identity to the 16S rRNA gene of the type-strain as a species cut off [57, 58, 74]. Not only were all 16S rRNA-based identifications invalidated by WGS-ANI results, but these differences were at times quite substantial. The most extreme cases being comparisons of isolates to the type strains of *Shewanella fidelis* (ATCC BAA-318), *S. loihica* (PV-4), *Vibrio profundi* (TP187) and *V. atlantis* (CECT 7223), which had respective WGS-ANIs of 85, 90, 86, and 89 %, despite the isolates having been confidently assigned to these species by 16S rRNA analysis. Poor 16S rRNA taxonomic identification has also been observed in other studies involving *Vibrio* species [75–77]. While results are noted here for reference, the 16S rRNA identifications of the 15 unidentified isolates were subsequently disregarded.

**Fig. 3.**
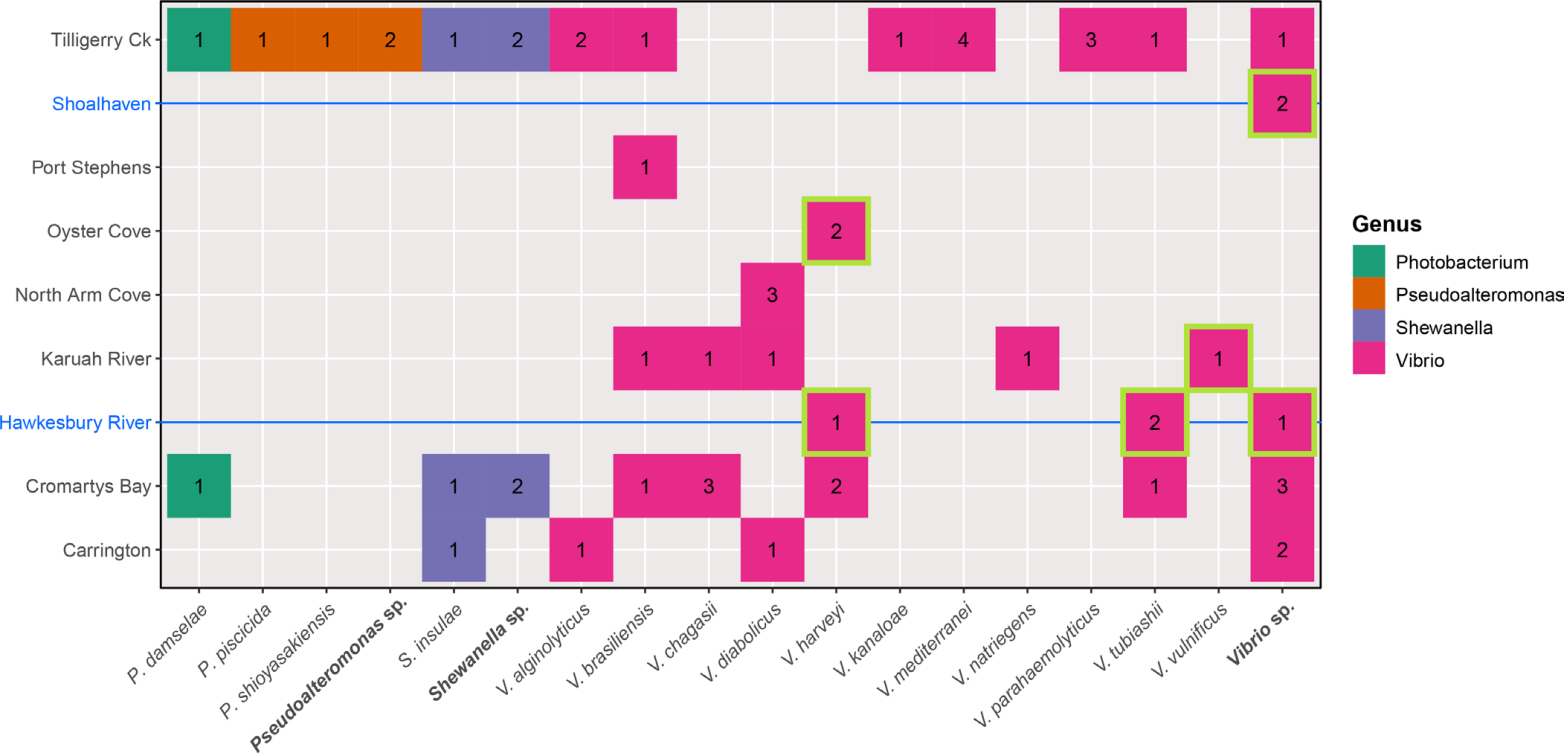
Number of isolates per bacterial species, obtained from POs grown at various locations around Port Stephens, or within the Hawkesbury River or Shoalhaven (the latter two marked with blue text and line). The isolates bordered in light green were not obtained from the 2013–2014 PS study but from PO leases within the Hawkesbury River, Shoalhaven, or Oyster Cove (PS). For each genus, the isolates belonging to unidentified species have been summed into one column.

**Fig. 4.**
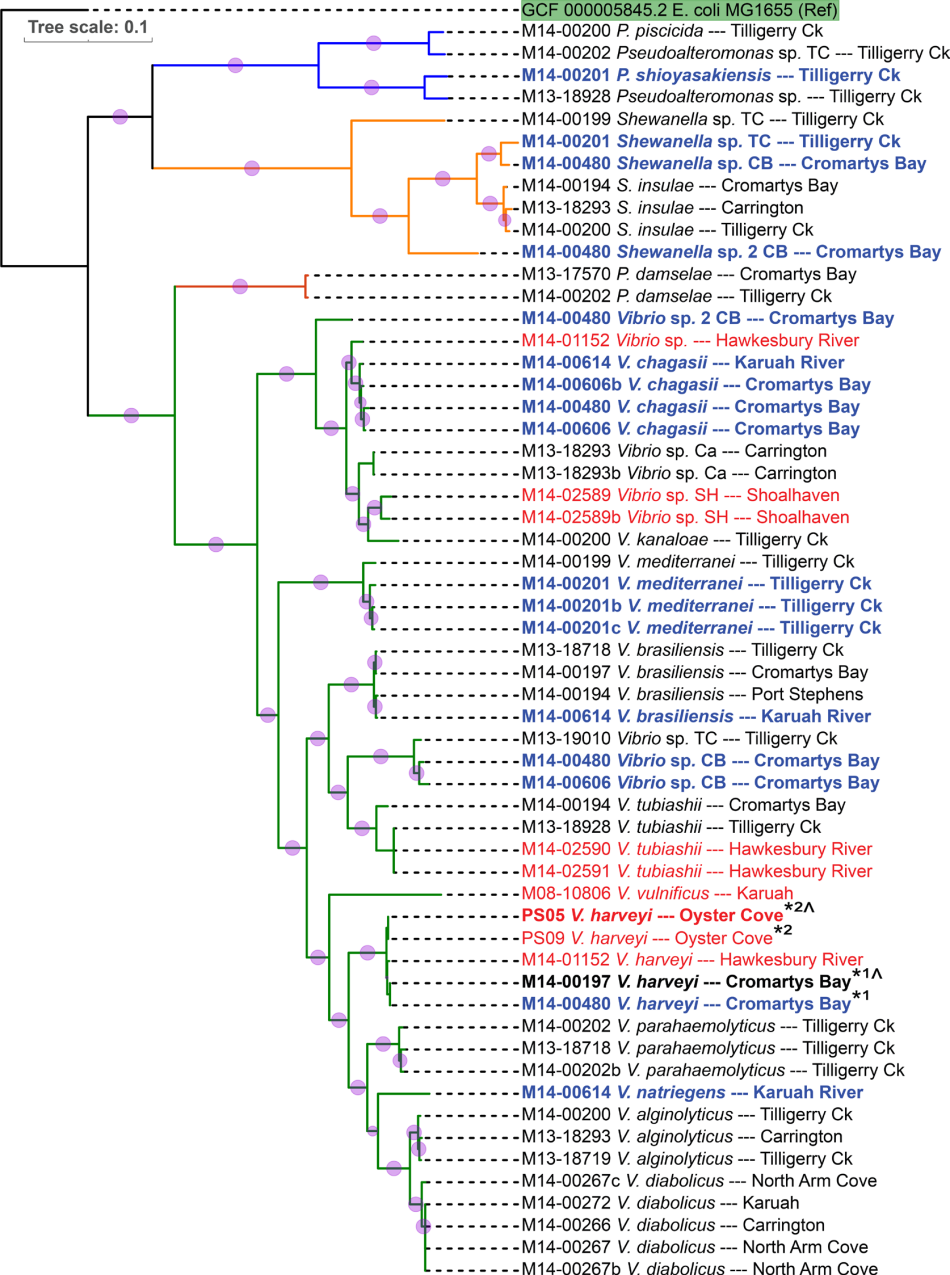
Phylogenetic tree constructed from selected core genes of the whole genome sequenced bacterial isolates used in this study. The genome alignment and tree construction were performed by the phylosift and FastTree programmes respectively, with an *E. coli* Genbank reference as the outgroup. The different genera are represented by different coloured branches and bootstrap values are proportional to the radius of each pink circle (largest=1). The isolate names marked in red text were not part of the 2013–2014 study, while the isolates in blue text originate from SM-unaffected POs within PS with black text representing SM-affected POs. The *V. harveyi* isolates marked with a single black asterisk are two sets of isolates, with each set of two isolates being virtually clonal except for the presence or absence of an 86 Kbp DNA fragment (suspected plasmid). From these two sets the two isolates containing a 86 Kbp fragment are in bold and marked with a carat (^). Numbers preceding the species or genus name represent the submission number of the batch of diseased oysters received. Occasionally multiple isolates originated from a single oyster.

For the WGS based ANI identifications, there were a diverse number of species observed (Fig. 3). Aside from the 15 identified species, there was a total of nine unidentified and potentially new species (represented in Fig. 3 only by genera), that were only able to be identified to the genus level using ANI comparisons. These unnamed species were from the genera *Pseudoalteromonas* (one), *Shewanella* (three), and *Vibrio* (five). Just under half of the unidentified species were represented by only one isolate, while one unidentified *Shewanella* and two unidentified *Vibrio* species each had two isolates. Only one unidentified *Vibrio* species had three representative isolates (Fig. 2 and Fig. 4). In total, there were potentially 24 species (15 identified and nine unidentified), of which even the 15 identified species alone represented a diverse number of species (Fig. 3) in comparison to most other reported SM events [37, 38, 78, 79]. Of the 57 isolates from this study, all were members of the class *Gammaproteobacteria *, with the majority from the *Vibrio* genus (Fig. 3). However, this is largely due to the culture-based approach used in Go *et al*. [4] that likely favoured *Vibrio* growth (culture on marine salt blood agar). The number of species identified from the *Vibrio* genus using WGS, was at least four times greater than the next most numerous genus (* Shewanella *). However, it should also be noted that no species identified was numerically dominant. Both *Vibrio harveyi* and *V. diabolicus* had the highest number of isolates, but only with five isolates each, out of a total of 57.

**Fig. 5.**
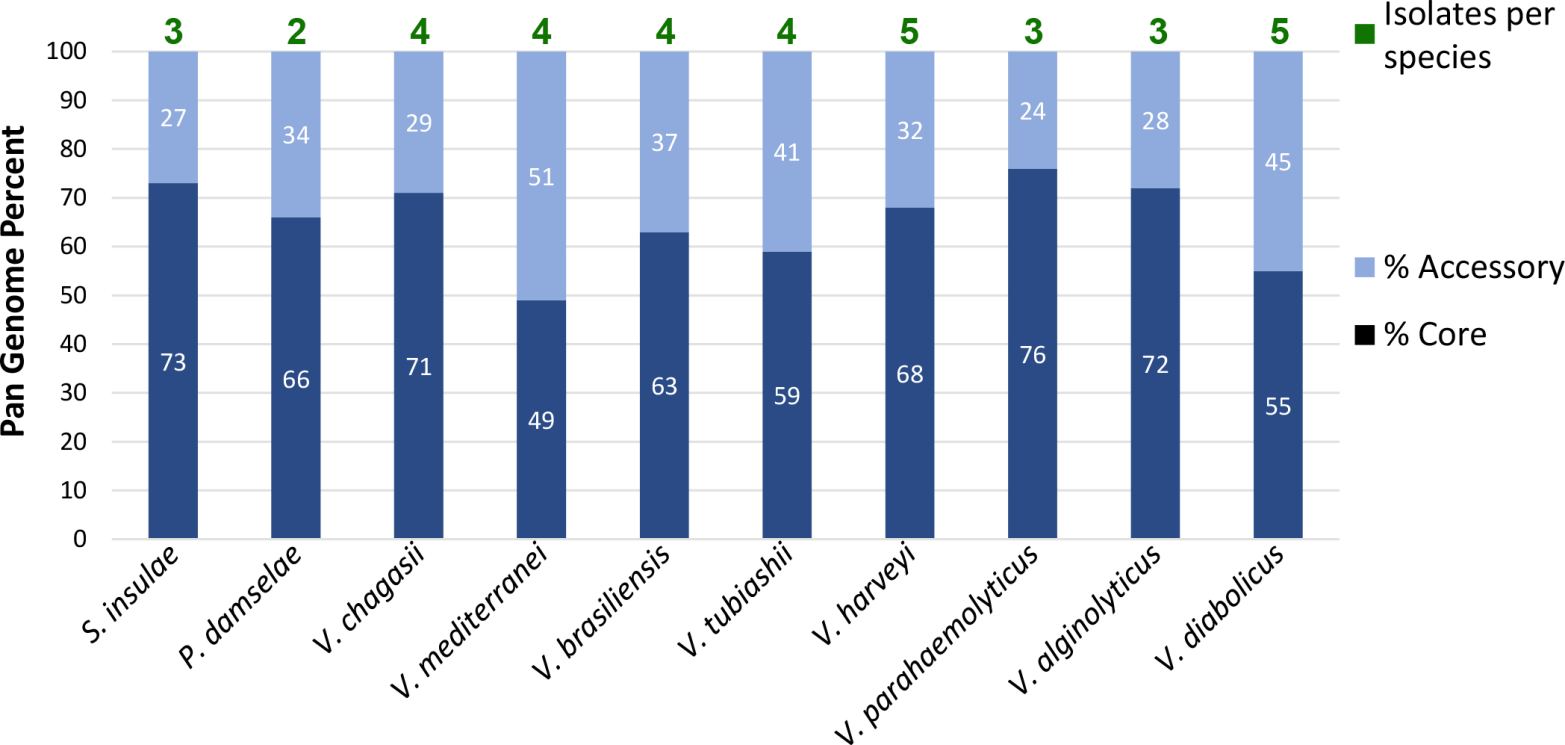
Summary of the pan-genome results for isolate species described in this study, in terms of both the core and accessory genomes that make up the pan-genome for each species.

To examine the genomic relationships between all 57 isolates and the species to which they belonged, a phylogenomic tree was constructed using the Phylosift and FastTree programmes (Fig. 4). As expected, the results showed two major branches split into the orders *Alteromonadales* and *Vibrionales *. The *Alteromonadales* branch split further into the *Pseudoalteromonas* and *Shewanella* genera, while the *Vibrionales* branch split into the genera *Photobacterium* (one species only) and *Vibrio* species. The taxonomic order of isolate species followed recent publications [80] and online taxonomic databases and visualisers (https://www.ncbi.nlm.nih.gov/taxonomy or http://lifemap-ncbi.univ-lyon1.fr/). Small differences in isolate clustering were observed between Figs 2 and 4, reflecting the different clustering algorithms used and the more information-rich data from which Fig. 5) Fig. 4 (multiple gene alignments) was derived.

The phylogenomic tree of Fig. 4 (and Figs 2 and 3), also provides insight into the diverse number of species, as well as the apparently large isolate diversity within each species, at least in terms of the geographical origin or the health-state (SM-affected or SM-unaffected) of the host POs. Of the ten identified species with multiple isolates, seven were obtained from POs grown at multiple locations, while only two species contained isolates originating from only one location (* V. mediterranei* and *V. parahaemolyticus *), although three of four *V *. *chagasii* also originated from one location. In contrast, there was a much greater association between species identification and the health state of the host. For the 17 isolates derived from unaffected POs, 11 belonged to species sets of two or more isolates while all four *V *. *chagasii* isolates originated from POs unaffected by SM, as did three of four isolates identified as *V. mediterranei *. Conversely, all the isolates of *V. diabolicus *, *V. alginolyticus *, *S. insulae *, and *Photobacterium damselae* were obtained from POs that had been affected by SM. To also investigate any gross functional diversity within isolates of the same species, a pan-genome analysis was performed on each identified species with three or more isolates. In particular, the accessory genome as a percentage of the pan-genome was examined (Fig. 5), as this is where adaptations to new environments or challenges are most likely to be found, and a likely indicator of functional diversity. Contrary to expectations, we observed that the accessory genome size for each species appeared not to be associated with that species’ geographic spread, nor whether they were obtained from affected vs unaffected oysters. For example, *S. insulae* had a small accessory genome (27 %), but its three corresponding isolates were each found at a different location, while *V. diabolicus* - with a relatively large (45 %) accessory genome – also had isolates from multiple locations, spaced many kilometres apart. Similarly, *V. parahaemolyticus* with the smallest accessory genome and *V. tubiashii* with one of the larger accessory genomes, were both represented by isolates that were all obtained from POs affected by SM. Of course, the small number of isolate genomes per species and thus the potential for random bias should also be considered.

**Fig. 6.**
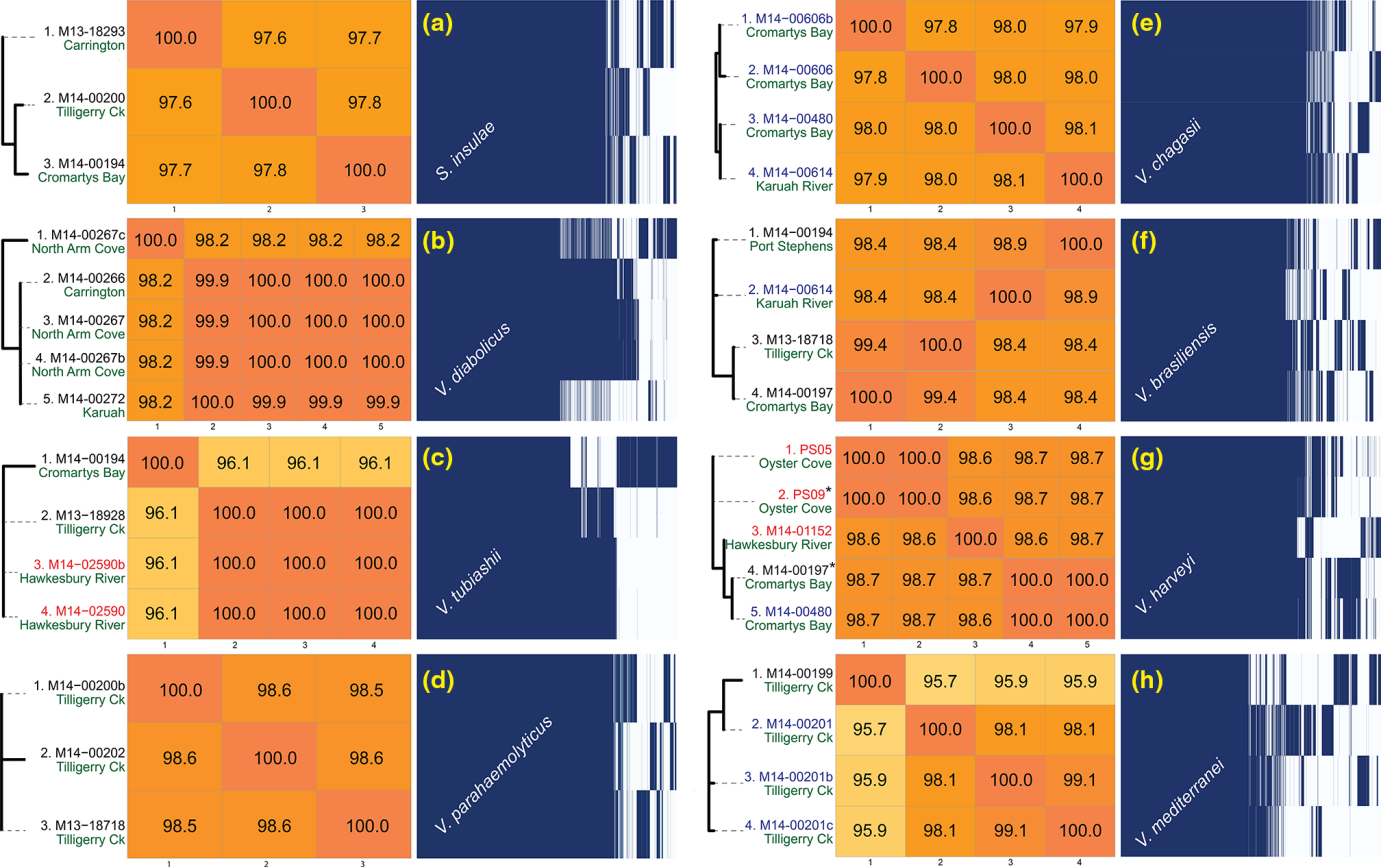
Side by side comparisons of ANIm percent identity with pan-genomes of selected isolates grouped into their species. Red text denotes isolates obtained from POs not part of the PS 2013–2014 SM study. Blue text denotes isolates taken from healthy POs unaffected by summer mortality during 2013–2014. The phylogenomic trees are only general representations of taxonomic distance between the isolate pan-genomes within a species. The two *V. harveyi* isolates marked with an asterisk, are both from a PO affected by summer mortality and contain an 86 Kbp DNA plasmid.

The above-mentioned correlations between species identifications and geographic location or health state of the host POs, the pan-genome of each species and the associated ANI of the isolates for each species were investigated (summarised in Fig. 6). Looking at the ANI results alone, only *S. insulae* (Fig. 6, section a) had differing ANI values corresponding to differing geographic origins, while *V. parahaemolyticus* (Fig. 6, section d) was the sole species with isolates of highly homologous sequences (ANI≥98.5 %), all originating from the same location. In juxtaposition, three of five *V *. *diabolicus* (Fig. 6, section b) and three of four *V *. *tubiashii* (Fig. 6, section c) isolates were extremely homologous in terms of ANI but had geographically dissimilar origins. It should be noted here that while two of the *V. diabolicus* (M14-00267 and M14-00267b) and two *V *. *tubiashii* (M14-02590 and M14-02590b) isolates had almost identical ANI results that correlated with the same geographical origin, both isolate pairs came from a single submission and were likely grown in proximity. In contrast to the limited and inconsistent associations to geographic origins, there were clearer associations between isolate ANI scores within each species and their relation to whether the isolates originated from SM-unaffected or SM-affected POs. All *V. chagasii* and three out of four *V *. *mediterranei* isolates were all highly homologous and from unaffected POs, with the remaining and most genetically distant *V. mediterranei* isolate having the lowest ANI score. Similarly, for *V. brasiliensis *, if both the ANI and pan-genome analyses were combined, the most genetically distant isolate was the only one (out of four) that originated from a PO unaffected by SM (see also Fig. 4). Moreover, when the pan-genome of each species was examined, there was clear evidence of diversity between the isolates of each species. In particular, the accessory genomes of isolates identified as *V. diabolicus *, *V. tubiashii *, *V. brasiliensis *, and *V. harveyi* displayed substantial variation in contrast to the extreme homology evident in the high ANI scores within each species. High diversity was observed in the accessory genomes of three of four *V *. *diabolicus* (Fig. 6, section b), three of four *V *. *tubiashii* (Fig. 6, section c), and four of five *V. harveyi* (Fig. 6, section g) isolates despite the virtually identical ANI scores (ANI≥99.9 %). Indeed, two sets of *V. harveyi* isolates (M14-00197, M14-00480 and PS05, PS09) were virtually identical (by ANI) but nonetheless displayed differences in their accessory genomes that were associated with whether the isolate was obtained from SM-affected or SM-unaffected POs (Fig. 6, section h).

Initial whole genome alignments of the WGS results via the progressive mauve package within the Geneious programme, led to the discovery of two virtually identical *V. harveyi* isolates: one originating from SM affected POs (M14-00197) and the other from SM unaffected POs (M14-00480). These isolates were almost entirely differentiated by the presence of an ~86 Kbp DNA fragment only present in the isolate (M14-00197) obtained from a SM affected PO. BLASTn-megablast searches confirmed the 86 Kbp DNA fragment to be homologous to several *Vibrio* plasmids that were themselves suspected of conferring pathogenicity, including plasmids from pathogenic strains of *V. campbellii* and *V. parahaemolyticus *. Indeed, annotation of this fragment by RAST and Prokka led to the identification of several potential virulence genes, such as: Type IV secretory pathway, VirB4 components CDS; Type IV secretory pathway, VirD4 components CDS and Recombinase. The 86 Kbp fragment was later confirmed to be circular via a PCR that spanned the fragment gap (OVP2b: Supplementary material, Table S1). The plasmid-containing and plasmid-less isolate were obtained from a single lease at Cromartys Bay, Port Stephens. Similarly, during this analysis two *V. harveyi* isolates with highly similar characteristics came to light from a separate study of POs from Oyster Cove, Port Stephens, which were subjected to a simulated marine heatwave [42]. We found that the putative pathogenic isolate (* V. harveyi* PS05) and the commensal isolate (* V. harveyi* PS09) from this study were also virtually clonal and again differentiated almost entirely by an ~86 Kbp plasmid found only in the isolate (PS05). Both *V. harveyi* PS05 and PS09 had been obtained from PO hosts that had been part of a study where POs were subjected to a simulated summer marine heatwave. The ~86 Kbp plasmid from M14-00197 and the ~86 Kbp plasmid from PS05 ware highly homologous with a 99.2 % pairwise identity. The distance between Cromartys Bay entrance (M14-00197) and Oyster Cove (PS05) is approximately 11 km by sea.

To further investigate whether other MGEs may be harbouring virulence-associated genes, a survey of MGEs was performed on the genomes of the *V. harveyi* isolates M14-00197, M14-00480, PS05 and PS09. In almost all cases many MGEs or their remnants were discovered in relatively equal numbers in all four genomes (supplementary material, Fig. S1). The exception being PS09 which had roughly half the number of repeat regions detected and three-fifths the number of insertion sequences. The numbers of MGEs detected are summarised in the supplementary material, Fig. S1 and Table S3. Additionally, as described above, the 86 Kbp plasmid was only found in the two *V. harveyi* isolates (M14-00197 and PS05) obtained from affected oysters and not the related strains M14-00480 and PS09 obtained from unaffected POs.

Selective gene ontology investigations were also performed, to examine whether any phenotypic differences between the isolates of a particular species might be related to apparent differences (or similarities) in the health state or geographic location of the host POs from which the isolates were obtained. We found that the accessory genome of *V. parahaemolyticus* (all potential pathogens obtained from the same site) included several gene ontology identifiers that are believed to be associated with MGEs, including functions such as plasmid maintenance and resistance to antibiotics and heavy metals,or MGE movement within and between genomes, including transposition; establishment of integrated proviral latency, and DNA-mediated transformation. However, a similar picture was seen for *V. chagasii *, *V. brasiliensis *, *V. harveyi *, and *V. mediterranei *, with each per species group of isolates differing in either location of origin (* V. chagasii *) or by potential pathogen versus commensality (* V. mediterranei* and *V. harveyi *). For completeness *V. brasiliensis* and *V. harveyi* were also included (differing by both origin of location and potential pathogen/commensal). Subsequently, a more focussed approach was used, where for each species, a subset of genes was obtained that were all present in one or more isolates representing a certain condition, but all absent in the comparative set. For example, genes present in all three *V *. *chagasii* isolates from Cromarty’s Bay but absent in the isolate from Karuah River. This also included *V. brasiliensis* and *V. mediterranei *, as well as four of five *V *. *harveyi* isolates where two isolates from Cromartys Bay were compared to one another (potential pathogen versus commensal), as were two from Oyster Cove. While gene ontology terms were again observed (including xenobiotic transmembrane transporter activity, bacterial-type flagellum, protein secretion systems, motility, pilus assembly, and oxidative stress response) the general result was just a reduced set of the same or similar terms. *V. mediterranei* was an exception amongst this group, possibly due to its larger accessory genome (Fig. 5 and Fig. 6) resulting in many terms being retained in the subset. To examine whether pan-genome components may be associated with phenotype, *V. chagasii* (location of origin), and *V. brasiliensis *, *V. harveyi *, and *V. mediterranei* (potential pathogenicity) data were input into Scoary, but no association was found.

## Discussion

POs (*C. gigas*) regularly experience large stock losses from a condition known as SM, with increasing evidence that implicates the involvement of opportunistic or pathogenic marine bacteria after one or more stress events [81–84]. The work described here targets SM events in POs that took place at the Port Stephens estuary in NSW, Australia, between January 2013 to January 2014 as outlined in Go *et al*. [4]. We sought to accurately identify the species and strains of the isolates that may have a role in SM by genomically characterising isolates obtained from the PS outbreak and subjecting their genomes to WGS, and subsequent genomic, phylogenomic, and pan-genomic analysis. As expected from the initial 16S rRNA gene-based species identifications from Go *et al*. [4], WGS of isolates and their ANI comparisons to reference genomes identified most isolates as belonging to the *Vibrio* genus. A total of 15 species of bacteria within the class *Gammaproteobacteria* were identified, including 11 *Vibrio* species. In addition to these 15, there were potentially an additional nine new species, which included five within the *Vibrio* genus. To our knowledge, this study represents the largest and most diverse genome survey of *Vibrio* species obtained from SM events in POs within the southern hemisphere, with the genomic data also serving as a comparative reference against future SM outbreaks in Australia. In total the results suggested that most species identified in this study were highly diverse, which was particularly evident in each species’ pan-genome. Indeed, there appeared to be a difference in the banding patterns between (isolate) accessory genomes of the same species when comparing the health state of the host POs (SM-affected or SM-unaffected).

A diverse group of species were identified from the 57 isolates examined in this study and included representatives from the *Photobacterium* (one), *Pseudoalteromonas* (two), *Shewanella* (one), and *Vibrio* (11) genera. *Vibrio* species were by far observed to be the most numerically dominant (Figs 2–4), and diverse genus. These results also mirror others that found large increases in *Vibrio* numbers within host POs during SM outbreaks. However, our results contrast with many studies of SM in POs that report only one or two dominant *Vibrio* species in affected oysters [6, 27, 78, 85, 86], although some European studies have also observed the possible involvement of multiple *Vibrio* species in SM [28–30]. Similarly, while a proportional increase in *Vibrio* species is often reported within POs infected by OsHV-1 (and its variants), these are often represented by only one or two dominant species [6, 25, 27, 28].

As also observed in other studies involving *Vibrio* (and *Shewanella *) species, 16S rRNA sequence analysis was found to be a poor tool for species identification [75, 76, 87–89]. This is unsurprising given the greater volume of information delivered by WGS, but it may also suggest that despite extensive characterisation of *Vibrio* genomic diversity there remains more to be discovered. Certainly, the *V. splendidus* clade is known to form a diverse phylogenetic cluster probably due to extensive lateral gene transfer [89], and *Vibrio* and *Shewanella* 16S rRNA gene sequences have been shown as inadequate at resolving individual *Vibrio* and *Shewanella* species [75, 76, 87, 88]. Indeed, WGS and other molecular techniques including the examination of housekeeping gene sequences [77] and other (non-WGS) genome-wide approaches [90–94] have demonstrated that the *Vibrio* genus is not only highly genetically diverse but has populated a plethora of different niche environments.

While the depth of *Vibrio* diversity remains to be fully understood, it is well known that many marine pathogens come from the *Vibrio* genus and of all the species vibrios are the most numerous in this study. Mortalities in clams have been reportedly caused by *V. alginolyticus* [95], while *V. parahaemolyticus* has been observed as a fish pathogen [96] as well as a pathogen in humans [97]. Similarly, *V. vulnificus* has also been observed as a human pathogen [98]. Transmission experiments have also shown that *V. brasiliensis *, *V. kanaloae* and *V. tubiashii* all cause disease in rainbow trout and brine shrimp [99]. In field observations, *Vibrio tubiashii* and *Vibrio coralliilyticus* have been observed to be highly infectious to eastern oysters [100], and *V. mediterranei* has been reported as a pathogen in pen shells [101] as well as coral [102]. In POs, *V. kanaloae* in association with *V. pomeroyi *, has been observed as a pathogen [79]. In scallops (*Patinopecten yessoensis*), mortality has been associated with *V. chagasii* [103], while *V. harveyi* has been associated with disease in many marine invertebrates [104]. *V. chagasii* (under the umbrella of *V. splendidus *), has been associated with mortalities of larval flat-fish (*Scophthalmus maximus*) [94], blue mussels (*Mytilus edulis*) [105], oysters (*C. gigas*) [28, 106] and carpet shell clam (*Ruditapes decussatus*) [95]. In contrast, *V. diabolicus* has not been observed as a pathogen in any marine animals. In short, many *Vibrio* species are known or potential pathogens, with some of these species, such as *V. harveyi* and those of the *V. splendidus* clade (that includes *V. chagasii *), also recognised as having a high genetic diversity [89, 107]. Other species or genera detected in this study are also suspected or known marine pathogens. *Photobacterium damselae *, is a potential fish, crustacean, and (for those exposed to it) human pathogen [108]. *Pseudoalteromonas shioyasakiensis* has been reported as an opportunistic pathogen in juvenile Pacific abalone (*Haliotis discus hannai*) [109]. In contrast, while *P. piscicida* has no known pathogenicity, it is known to form biofilms with antimicrobial capacity and produce several toxin genes [110]. Similarly, *S. insulae* [111] has no known pathogenicity, although other *Shewanella *, namely *S. putrefaciens* and *S. algae* are human pathogens [112]. Thus, it is conceivable that some of the species described by this study have a highly diverse pan-genome, capable of acting as a genomic scaffold upon which new strains can evolve via horizontal gene transfer and gene capture [89, 113, 114]. In this scenario, genes or whole sections of genomes could potentially be shuffled around (for example, via mobile genetic elements) until a new combination evolves that results in niche success (including pathogenesis).

Our results also suggest (some) *Vibrio* species are highly diverse and that identification of *Vibrio* pathogens should probably be at the taxonomic level of strain. For almost all the *Vibrio* species within the phylogenomic tree of Fig. 4, their isolates were obtained from host POs that were many kilometres apart and/or had differing states of health (SM-affected or SM-unaffected). Species diversity was even more apparent from the ANI results, which for most *Vibrio* species identified, juxtaposed the high sequence similarity within isolates of the same species to the differences in geographical origin and/or health state (SM-affected or SM-unaffected) of the PO host from which the isolate was obtained. Certainly, from Fig. 6, only *V. mediterranei* isolates displayed any association between ANI scores and the health of their host POs. In comparison subgroups of isolates from *V. diabolicus *, *V. tubiashii *, and *V. harveyi* had extremely high ANI scores (minimum ANI of 99.9 %.) despite their differing host origins and health states. Species identification for *Vibrios* as a limited pathogenicity indicator has been suggested in several studies [85, 113, 115].

In contrast to the sequence comparison by ANI, a function-based analysis was effective at revealing the genomic diversity within isolates of the same species. For the virtually identical isolates (ANI >99.9 %) within the *V. diabolicus *, *V. tubiashii *, and *V. harveyi* species (Fig. 6, parts b, c, and g), pan-genome analysis was able to detect differential banding patterns in the accessory genome that not only reflected individual isolates but also the similarities corresponding to the same geographic origin of the host PO or the health state of the PO (SM-affected versus SM-unaffected). This was also seen in all other species with multiple isolates for comparison, including the two pairs of *V. harveyi* isolates (M14-00197 and M14-00480; PS05 and PS09) that each contained two isolates of extreme homology (ANI ≥99.99 %). Indeed, the differences in the accessory genes within the two sets of virtually sequence identical *V. harveyi* isolates correspond with the presence of an 86 Kbp plasmid in one isolate of each set (M14-00197 and PS05), and its absence in the corresponding (commensal) isolate of each set (M14-00480 and PS09). Moreover, both *V. harveyi* isolates with an 86 Kbp plasmid (M14-00197 and PS05) were obtained from their respective host POs that were each 11 km apart, yet in terms of length, sequence, and annotated genes the two plasmids were almost identical. This included several virulence factors that mapped to both plasmids, such as: Type IV secretory pathway, VirB4 components; Type IV secretory pathway, VirD4 components; and recombinase. In addition, for all these four *V. harveyi* isolates there was evidence of many MGEs scattered throughout the genomes at roughly similar numbers (supplementary material, Fig. S1). MGEs such as insertion sequences, prophage sequences, and inverted repeats at similar levels suggested that these four isolates once had, or still retained, the capability of shuffling genetic material within their own genomes, or between genomes of other strains or species. Moreover, we also found from the accessory genomes of *V. parahaemolyticus *, *V. chagasii *, *V. brasiliensis *, *V. harveyi,* and *V. mediterranei *, numerous gene ontology terms that are suspected of being involved with MGEs (supplementary material, Table S4 and Figs S2–S4).

## Conclusion

Here we have genomically characterised a group of bacterial isolates obtained from POs affected by SM events occurring between January 2013 and January 2014 at Port Stephens, New South Wales. Not only were many marine bacterial species observed, including many from the *Vibrio* genus, but phylogenomic, sequence and pan-genome analysis in combination with metadata also suggested that most of the identified species have highly diverse genomes. While we were not able to definitively link a single species with SM, associations between isolate accessory genomes and the health state of their hosts were evident. We found two instances, in *V. harveyi* strains, where these differences were attributable to the presence of plasmids. In summary, the large number of species detected during the SM outbreak, the within-species diversity and the presence of virulence-associated genes in a couple of strains, suggest that many different *Vibrio* species could have the capacity to acquire pathogenicity under adverse conditions.

## Supplementary Data

Supplementary material 1

Supplementary material 2
