# Supplementary material for: Phylogenomic diversity of Vibrio species and other Gammaproteobacteria isolated from Pacific oysters (Crassostrea gigas) during a summer mortality outbreak
Source: Microb Genom. 2022 Dec 7;8(12):mgen000883. doi: 10.1099/mgen.0.000883 (PMC9837568; doi:10.1099/mgen.0.000883)
Supplement: Supplementary material 1 [file mgen-8-883-s001.pdf]

Table S1. Primers used to confirm presence of 86 Kbp *V. harveyi* plasmid

Primer name	Primer sequence 5'-3'
OVP2bF	AATTTCACCTGACCGACCGACGAT
OCP2bR	CAGGAAGAATTGGTAGAGGTAAGC

Table S2. Quast summary of Port Stephens isolates

Name	No. contigs	Largest contig	Total length (kbp)	GC (%)	N50	N75	L50	L75	Ns per 100
M13-18293b Vibrio Sp. Ca --- Carrington	88	344,395	4,692,430	44.32	110,779	74,875	14	26	7.46
M14-00480 Shewanella Sp. CB --- Cromartys Bay	18	2,154,121	4,508,292	53.01	867,182	744,438	2	3	0.98
M14-00606 V. splendidus --- Cromartys Bay	27	1,299,687	5,311,263	44.46	404,226	263,704	4	8	4.63
M13-18293 S. insulae --- Carrington	33	597,273	4,548,405	52.95	246,072	169,993	6	12	3.5
M14-00200 S. insulae --- Tilligerry Ck	67	403,872	4,431,966	53.08	170,190	78,618	9	19	0.72
M14-02590 V. tubiashii --- Hawkesbury River	18	2,252,541	5,437,082	44.97	1,743,904	350,790	2	3	1.4
M13-18928 P. donghaensis --- Tilligerry Ck	286	174,287	4,547,596	40.78	31,604	17,917	43	91	1.25
M14-00201 V. mediterranei --- Tilligerry Ck	290	191,430	6,571,930	43.96	45,206	26,336	44	91	1.98
M14-00197 V. brasiliensis --- Cromartys Bay	215	166,765	5,267,894	45.9	48,791	25,300	37	75	1.5
M14-00480 Vibrio Sp. CB --- Cromartys Bay	23	1,854,726	4,776,348	44.69	969,910	880,640	2	3	4.79
M14-00202 V. parahaemolyticus --- Tilligerry Ck	23	2,272,268	4,995,260	45.38	1,161,470	585,712	2	3	3.28
M14-00200 V. kanaloae --- Tilligerry Ck	154	193,718	4,698,411	43.81	66,604	33,942	24	48	1.04
M14-00202 Pseudoalteromonas Sp. TC --- Tilligerry Ck	433	73,709	5,194,877	43.12	21,018	11,298	69	156	2.08
M14-00201b V. mediterranei --- Tilligerry Ck	690	82,152	6,019,004	44.18	16,057	8,681	111	239	5.05
M08-10806 V. vulnificus --- Karuah	62	713,493	5,144,648	46.26	466,731	147,902	5	9	6.8
M14-00200 V. alginolyticus --- Tilligerry Ck	414	135,493	5,458,104	44.53	47,917	24,402	34	74	8.04
M14-00199 Shewanella Sp. TC --- Tilligerry Ck	83	278,902	4,910,282	45.61	113,577	62,950	14	28	1.83
M14-00201c V. mediterranei --- Tilligerry Ck	22	1,163,623	5,576,467	44.09	881,932	331,000	3	6	3.1
M14-00194 V. tubiashii --- Cromartys Bay	178	216,534	5,751,657	44.77	57,311	31,952	31	64	1.51
M14-00480 V. splendidus --- Cromartys Bay	98	313,060	5,471,227	44.42	142,529	65,076	13	27	1.37
M14-00606 Vibrio Sp. CB --- Cromartys Bay	34	1,548,716	4,762,806	44.69	285,736	123,568	3	9	3.59
M14-00614 V. splendidus --- Karuah River	44	1,118,369	5,450,824	44.38	307,692	186,508	6	11	4.31

M14-00267 V. diabolicus --- North Arm Cove	30	1,074,754	5,545,275	44.43	768,308	412,981	3	6	4.72
M14-00614 V. natriegens --- Karuah River	59	401,319	5,442,688	44.93	234,674	135,195	9	17	3.27
M14-00266 V. diabolicus --- Carrington	746	92,829	5,476,103	44.67	14,092	7,109	113	248	3.18
M14-00201 Shewanella Sp. TC --- Tilligerry Ck	220	163,741	4,549,217	52.73	49,222	25,068	32	63	5.1
M13-18293 V. alginolyticus --- Carrington	39	812,343	5,119,150	44.59	403,032	158,211	5	10	4.77
M14-00200 P. piscicida --- Tilligerry Ck	392	122,835	5,444,733	43.29	25,258	13,616	62	135	2.98
PS05 V. harveyi --- Oyster Cove	4	3,629,035	5,973,111	44.79	3,629,035	2,207,530	1	2	2.98
M13-18718 V. parahaemolyticus --- Tilligerry Ck	136	341,882	5,138,243	45.36	83,036	40,187	20	41	2.96
M14-00606b V. splendidus --- Cromartys Bay	48	632,346	5,491,378	44.45	362,176	173,190	6	11	1.26
M14-00614 V. brasiliensis --- Karuah River	20	1,516,374	4,861,909	45.63	590,724	253,489	3	6	2.61
M13-17570 P. damselae --- Cromartys Bay	60	430,010	4,743,095	40.41	196,357	126,701	8	15	9.23
M14-00202 P. damselae --- Tilligerry Ck	102	362,288	4,539,510	40.64	129,729	75,217	12	23	3.35
M14-00480 Vibrio Sp.2 CB --- Cromartys Bay	34	1,672,436	5,234,512	44.72	410,835	221,267	3	8	2.81
M14-00194 S. insulae --- Cromartys Bay	70	367,026	4,505,276	53.08	126,668	77,801	10	22	0.93
M13-19010 Vibrio Sp. TC --- Tilligerry Ck	35	1,578,158	4,859,961	44.74	521,719	214,195	3	7	2.78
M14-00480 V. harveyi --- Cromartys Bay	121	374,994	6,089,890	44.79	107,804	61,004	17	37	1.33
M14-00267b V. diabolicus --- North Arm Cove	50	1,044,197	5,568,509	44.43	530,939	230,367	4	8	1.26
M14-01152 V. chagasii --- Hawkesbury River	162	315,286	5,825,620	44.39	84,623	43,014	20	45	2.59
M14-00480 Shewanella Sp.2 CB --- Cromartys Bay	16	1,209,086	3,696,375	44.58	874,425	370,885	2	4	1.95
M14-00272 V. diabolicus --- Karuah	2742	12,054	4,665,587	45.63	2,191	1,274	639	1344	36.31
M14-02589b Vibrio Sp. SH --- Shoalhaven	66	575,257	4,926,843	44.44	205,704	142,531	8	15	2.6
M14-00202b V. parahaemolyticus --- Tilligerry Ck	30	1,792,606	5,141,381	45.27	1,116,173	510,658	2	4	1.54
M14-01152 V. harveyi --- Hawkesbury River	23	2,254,498	5,671,446	44.97	873,848	510,905	2	4	2.56
M14-00197 V. harveyi --- Cromartys Bay	79	374,993	6,133,066	44.73	145,532	83,027	14	28	3.55
M14-00199 V. mediterranei --- Tilligerry Ck	27	2,591,286	5,893,262	44.09	1,709,299	384,637	2	3	1.34
PS09 V. harveyi --- Oyster Cove	93	364,322	5,818,446	44.79	231,327	123,748	10	18	0
M14-00267c V. diabolicus --- North Arm Cove	83	538,395	5,337,082	44.59	184,058	78,056	11	23	1.82
M14-00194 V. brasiliensis --- Port Stephens	19	1,896,559	5,054,502	45.75	890,290	377,800	2	4	3.13

M14-00201 P. donghaensis --- Tilligerry Ck	321	163,638	4,746,171	41.12	26,879	14,140	51	109	4.89
M13-18293 Vibrio Sp. Ca --- Carrington	315	101,402	4,713,045	44.37	28,033	16,613	49	104	6.68
M13-18719 V. alginolyticus --- Tilligerry Ck	235	148,324	5,199,581	44.6	49,019	27,985	33	67	6.15
M14-02590b V. tubiashii --- Hawkesbury River	16	2,252,761	5,433,503	44.96	1,741,881	352,582	2	3	1.05
M14-02589 Vibrio Sp. SH --- Shoalhaven	45	786,057	5,381,065	44.56	217,151	141,861	6	14	1.95
M13-18718 V.brasiliensis --- Tilligerry Ck	46	823,942	5,327,112	45.85	248,022	170,371	6	12	2.16
M13-18928 V. tubiashii --- Tilligerry Ck	657	72,210	5,362,979	45.13	13,465	7,542	121	252	3.77

Table S3. Numbers of repeat regions and insertion sequences detected in *V. harveyi* isolate genomes

**Isolate**	**Isolate set**	**Number of repeat regions detected in genome**	**Insertion sequences**
M14-00197	1	721	31
M14-00480	1	642	30
PS-05	2	718	15
PS-09	2	301	9

Table S4. Selected gene ontology terms, potentially related to pathogenicity, summarised by Revigo and CirGO

**Species and accessory gene set description**	**Summarised GO terms with possible connection to MGEs**	**FOCUSSED SUBSET* - Summarised GO terms with possible connection to MGEs**
V. parahaemolyticus	Plasmid maintenance<br>DNA-mediated transformation<br>Transposition<br>Establishment of integrated proviral latency<br>Response to antibiotic	None
V. chagasii	Response to antibiotic<br>Establishment of integrated proviral latency<br>Pilus assembly	Chemotaxis
V. brasiliensis	Plasmid maintenance<br>Type IV pilus-dependent motility<br>Pilus assembly<br>Antibiotic catabolic process<br>Toxin activity<br>Toxic substance binding	SOS response<br>DNA integration
V. harveyi	Toxin activity<br>Toxin transmembrane transporter activity<br>Efflux transmembrane transporter activity<br>Pilus	DNA restriction-modification system<br>Protein secretion

	Type III protein secretion system complex<br>Type II protein secretion system complex<br>Regulation of bacterial-type flagellum assembly<br>Antibiotic catabolic process<br>Xenobiotic transport<br>Chemotaxis	
V. mediterranei	Toxin activity<br>Antioxidant activity<br>Type I protein secretion system complex<br>Type II protein secretion system complex<br>Bacterial-type flagellum<br>Cell adhesion<br>Viral capsid assembly<br>Xenobiotic transport<br>Type IV pilus-dependent motility<br>Pilus assembly	Antioxidant activity<br>Xenobiotic transmembrane transporter activity<br>Bacterial-type flagellum<br>Type I protein secretion system complex<br>Type II protein secretion system complex<br>Homophilic cell adhesion via plasma membrane adhesion molecules<br>Viral capsid assembly<br>Type IV pilus-dependent motility<br>Response to oxidative stress<br>Pilus assembly

*A subset of genes was selected from the accessory genome of a species as output by Roary based on the presence of genes in all isolates of one set and the absence of the same genes in all isolates of the comparative set

Mobile genetic elements in 4 *V. harveyi* isolate genomes

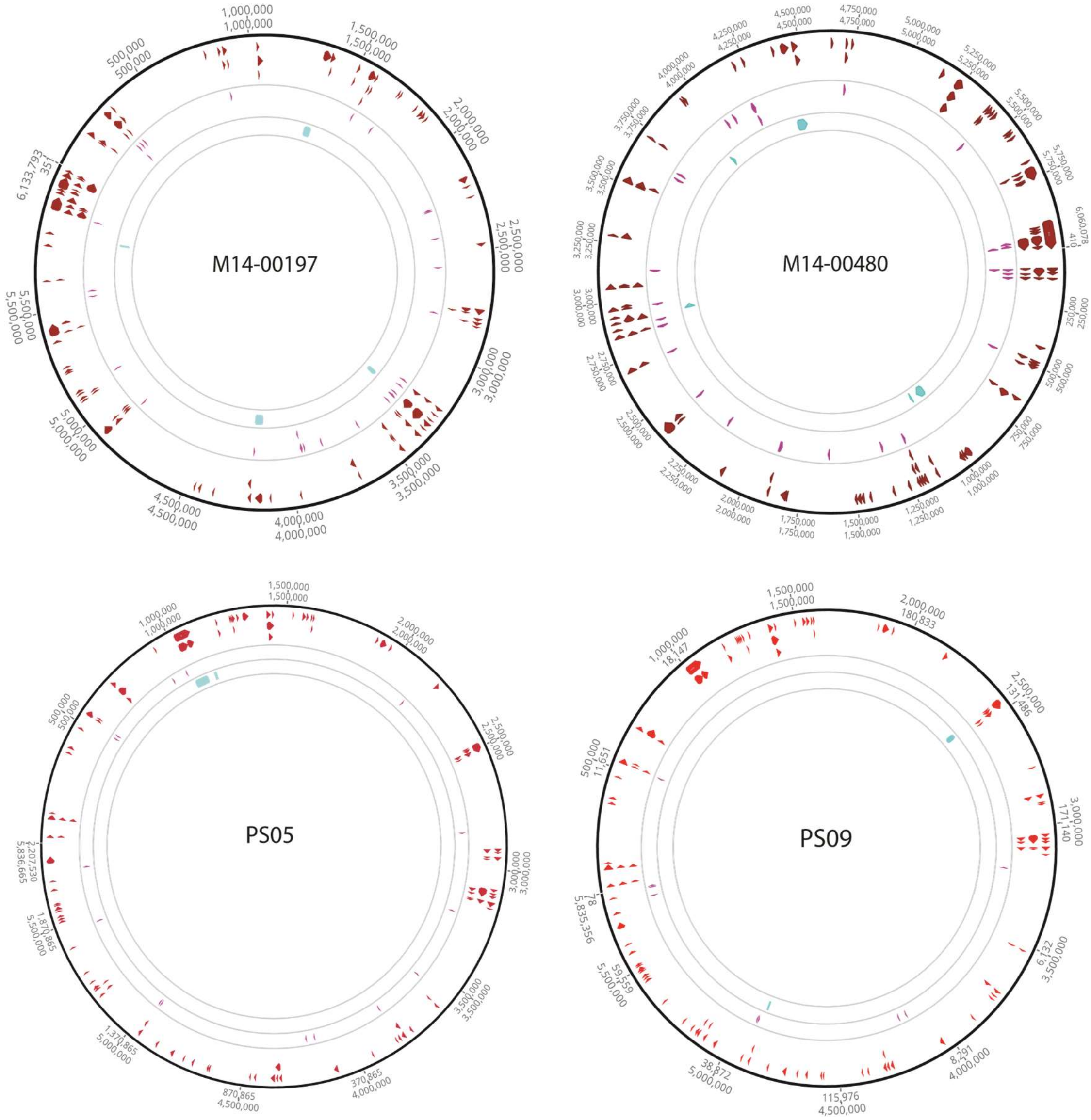

Figure S1. Mobile genetic elements within four *V. harveyi* genomes. Genomic islands are in red (outer track), insertion sequences are in pink (middle track), and prophage sequences are in light blue (inner track). Hundreds of Inverted repeat were detected in all genomes (see supplementary table B) but were too numerous to display, while two 86 Kbp plasmids were found in *V. harveyi* isolates M14-00197 and PS05 (not displayed). See Table S3 for other MGEs detected.

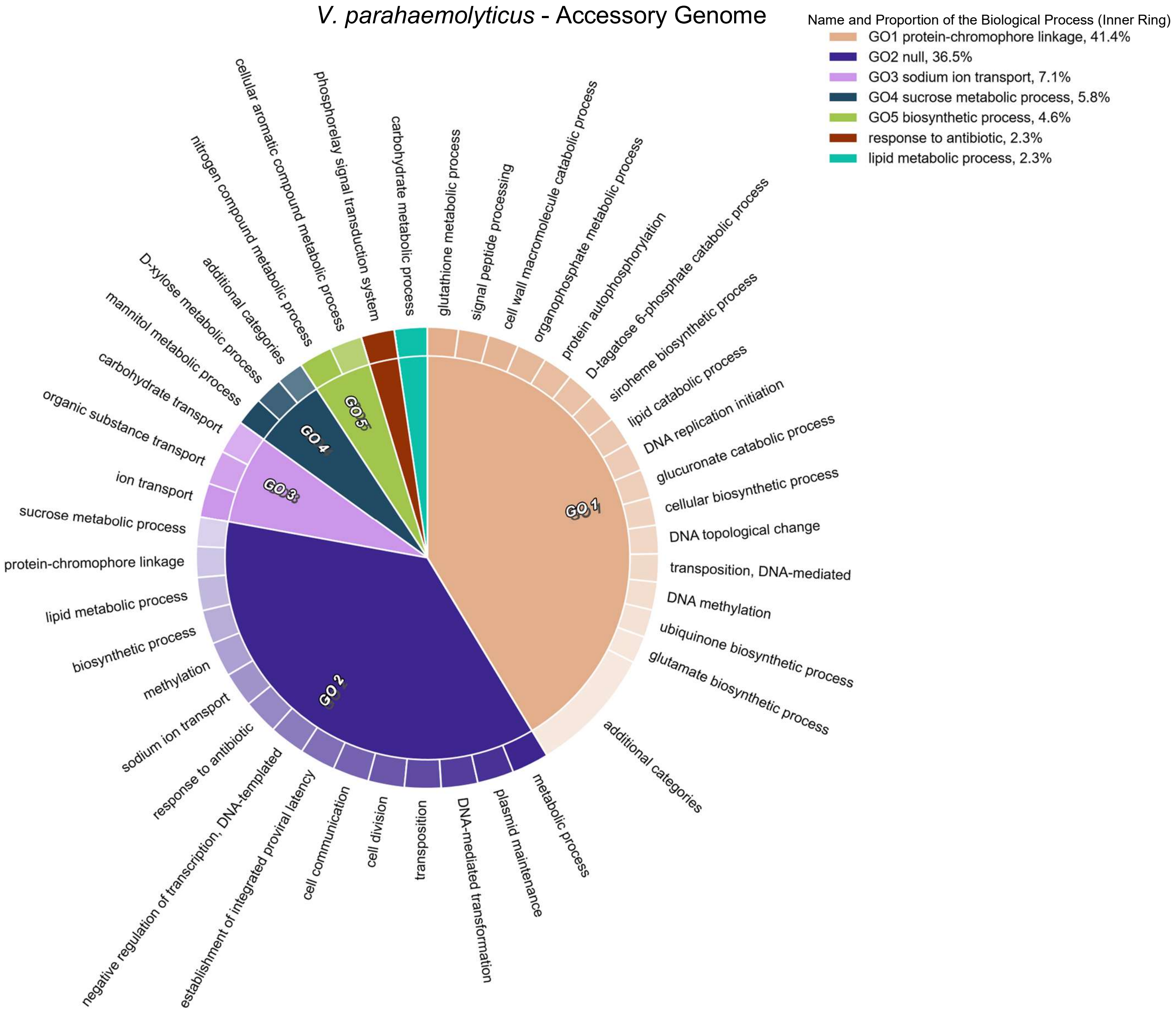

Figure S2. *V. parahaemolyticus* visual summary of gene ontology terms (Biological process parent category) for the accessory genome as determined by Roary. The plot was drawn by CirGO from data obtained from Revigo.

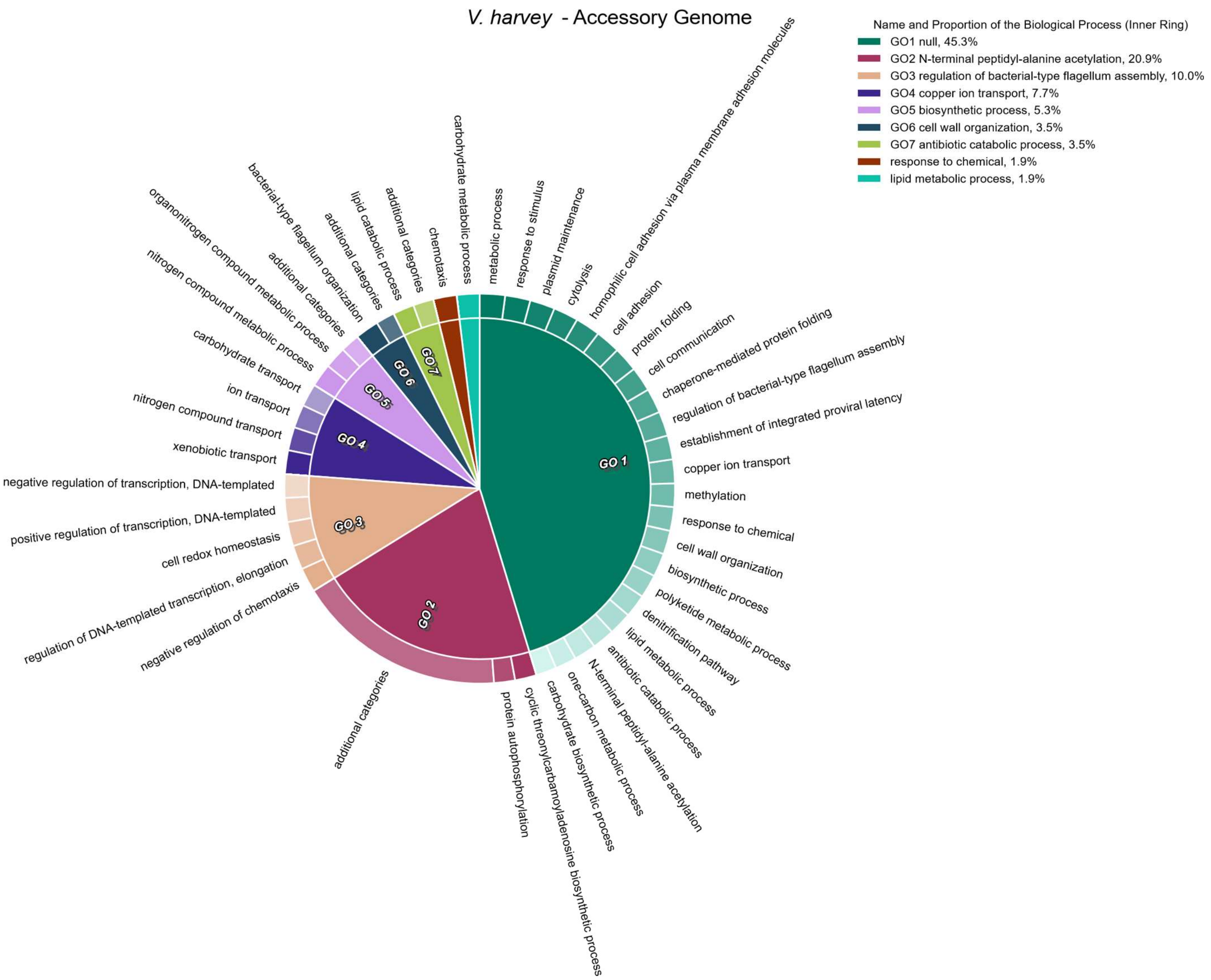

Figure S3. *V. harveyi* visual summary of gene ontology terms (Biological Process parent category) for the accessory genome as determined by Roary. The plot was drawn by CirGO from data obtained from Revigo.

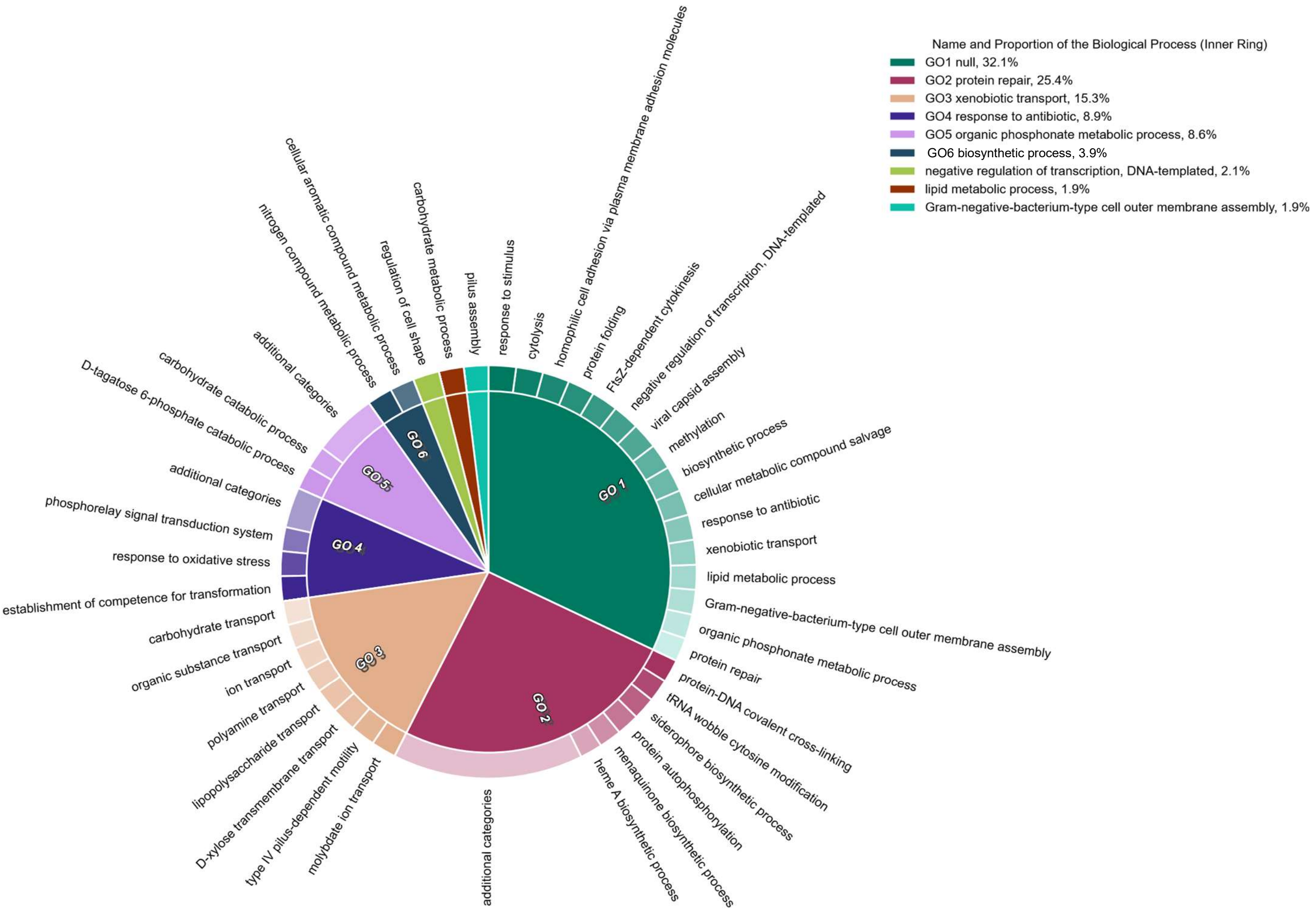

Figure S4. *V. mediterranei* visual summary of gene ontology terms (Biological Process parent category) focussed on genes for the accessory genome (Biological process parent category) as determined by Roary. The plot was drawn by CirGO from data obtained from Revigo.
